# Supplementary material for: Protocol to develop sustainable day care for children aged 1–4 years in disadvantaged urban communities in Dhaka, Bangladesh
Source: BMJ Open. 2018 Aug 1;8(7):e024101. doi: 10.1136/bmjopen-2018-024101 (PMC6074616; doi:10.1136/bmjopen-2018-024101)
Supplement: Author's manuscript [file bmjopen-2018-024101.draft_revisions.pdf]

BMJ Open is committed to open peer review. As part of this commitment we make the peer review history of every article we publish publicly available.

When an article is published we post the peer reviewers' comments and the authors' responses online. We also post the versions of the paper that were used during peer review. These are the versions that the peer review comments apply to.

The versions of the paper that follow are the versions that were submitted during the peer review process. They are not the versions of record or the final published versions. They should not be cited or distributed as the published version of this manuscript.

BMJ Open is an open access journal and the full, final, typeset and author-corrected version of record of the manuscript is available on our site with no access controls, subscription charges or pay-per-view fees (http://bmjopen.bmj.com).

If you have any questions on BMJ Open's open peer review process please email info.bmjopen@bmj.com

BMJ Open

# A protocol to develop a sustainable day-care for 1-4 year olds in disadvantaged urban communities in Dhaka, Bangladesh: a mixed methods study



| | |
|---|---|
| Journal: | *BMJ Open* |
| Manuscript ID | bmjopen-2018-024101 |
| Article Type: | Protocol |
| Date Submitted by the Author: | 15-May-2018 |





| | |
|---|---|
| Complete List of Authors: | Das, Mahua; Leeds University, Leeds Institute of Health Sciences<br>Elsey, Helen; University of Leeds, Nuffield Centre for International Health and Development<br>Shawon , R.A; Centre for Injury Prevention and Research Bangladesh ,<br>Hicks, Joseph; University of Leeds, Nuffield Centre for International Health and Development, Leeds Institute of Health Sciences<br>Ferdoush, J; Centre for Injury Prevention and Research Bangladesh<br>Huque, Rumana; University of Dhaka, Economics<br>Fieroze, Fariza; ARK Foundation<br>Nasreen, Shammi; ARK Foundation,<br>Wallace, Hilary; The University of Notre Dame Australia, School of Medicine,<br>Mashreky, Saidur; Centre for Injury Prevention and Research Bangladesh, |





| | |
|---|---|
| Keywords: | Protocols & guidelines < HEALTH SERVICES ADMINISTRATION & MANAGEMENT, Child protection < PAEDIATRICS, Community child health < PAEDIATRICS, PUBLIC HEALTH, QUALITATIVE RESEARCH |



SCHOLARONE™
Manuscripts

# A protocol to develop sustainable day-care for 1-4 year olds in disadvantaged urban communities in Dhaka, Bangladesh

Authors:

1. **Das, M.** Nuffield Institute for International Health and Development, University of Leeds, m.das@leeds.ac.uk ; Phone number: 0044-7809601932
2. **Elsey, H**. Nuffield Institute for International Health and Development, University of Leeds, h.elsey@leeds.ac.uk.
3. **Shawon, RA.** Centre for Injury Prevention and Research Bangladesh (CIPRB) riasha47@gmail.com
4. **Hicks, J**. Nuffield Institute for International Health and Development, University of Leeds, J.P.Hicks@leeds.ac.uk
5. **Ferdoush, J.** Centre for Injury Prevention and Research Bangladesh (CIPRB), junnatul.ferdoush@gmail.com
6. **Haque, R**. ARK Foundation, rumanah14@yahoo.com
7. **Fieroze, F**. ARK Foundation farizafieroze@yahoo.com
8. **Nasreen, S**. ARK Foundation, shammibadrul@yahoo.com
9. **Wallace, H**. The University of Notre Dame Australia, School of Medicine, Fremantle, hilary.wallace@nd.edu.au
10. **Mashreky, SR.** Centre for Injury Prevention and Research Bangladesh (CIPRB) mashreky@ciprb.org

**Word count**- 5,282 words excluding the abstract, tables, figures and references.



For peer review only - http://bmjopen.bmj.com/site/about/guidelines.xhtml

A protocol to develop of sustainable day-care for 1-4 year olds in disadvantaged urban communities in Dhaka, Bangladesh

**Abstract:**

**Introduction**: Lack of safe, stimulating and health-promoting environments for children under-5 hinders their physical, social and cognitive development, known as Early Childhood Development (ECD). Improving ECD not only impacts on children, but can improve educational attainment for girls, who often care for younger siblings, and employment prospects for mothers. Developing and evaluating the impacts of ECD programmes within childcare needs to assess a range of social, health, educational and economic impacts, including women's empowerment.

Children living in slums are at high risk of poor early development and holistic, sustainable interventions are needed to address ECD in these contexts. This study will be undertaken in Dhaka, Bangladesh, a city where over 8.5 million inhabitants live in slums. In collaboration with government, NGOs and communities, we are developing and testing a sustainable day-care model for low-income communities in Dhaka.

**Methodology and Analysis**:

A sequential mixed methods approach is being used in the study, with qualitative work exploring quantitative findings. Two hundred households with children under-5 will be surveyed to determine day-care needs and to assess ECD (parent reported and direct assessment). The feasibility of four ECD measuring tools Caregiver-Reported Early Development Index , Measuring Early Learning Quality and Outcomes, The Early Human Capability Index and International Development and Early Learning Assessment will be assessed quantitative and qualitatively. Qualitative methods will help understand demand and perceptions of day-care while mothers work. Participatory action research will be used to develop a locally appropriate and potentially sustainable model of day-care for under 5 children. A ward in the south of Dhaka has been selected for the study as this typifies communities with slum and non-slum households living next to each other, allowing us to explore potential for better-off household to subsidise day-care for poorer households.

**Ethics and dissemination**:

Findings will be published and inform decision makers at the national, regional and the local actors in order to embed the study into the policy and practice on childcare and ECD. University of Leeds Ethical Review Board approved: (ref: MREC16-106) and the Bangladesh Medical Research Council (ref: BMRCAIREC/20 I 6-20 I 9 I 250

**Keywords:** Urban slum, Day-care, Under-5 children, Early childhood development, Bangladesh, Low income countries

A protocol to develop of sustainable day-care for 1-4 year olds in disadvantaged urban communities in Dhaka, Bangladesh

**Strengths and weakness of the study**

**Strengths**:

1. Our participatory action research will provide valuable insights into how to design and deliver day-care that is accessible to poor urban households.
2. Our survey will fill a gap in the evidence by providing information on the demand for day-care among slum and non-slum households.
3. Continual engagement with policy makers and practitioners will help to explore options for scale-up of a sustainable model.

**Weaknesses**:

1. Due to the available resources we are only able to develop the day-care model in one community, potentially limiting the identification of delivery issues that might occur in other urban neighbourhoods.
2. Our questionnaire is only conducted in one ward of Dhaka. While our sample size is sufficiently powered to provide estimations of demand for day-care, the external validity of the questionnaire to make inferences to other types of neighbourhoods within Dhaka and other low-income cities is limited.



For peer review only - http://bmjopen.bmj.com/site/about/guidelines.xhtml

A protocol to develop of sustainable day-care for 1-4 year olds in disadvantaged urban communities in Dhaka, Bangladesh

# 1 BACKGROUND

Rapid urbanization, with over 60 per cent of the world's population is expected to live in cities by 2030 and nearly 70 per cent by 2050, is a major global concern and has led to a sustainable development goal focused specifically on sustainable cities [1]. Much of this growth is seen in low income countries where an increasing number of the urban population live in slum conditions[1] Such conditions increase exposure to communicable diseases and risk factors for non-communicable diseases increasing the risk of poor health, social and cognitive outcomes during childhood and later in life [2, 3]. With slum-dwelling families, particularly women, working long hours and limited availability of extended family, slum communities face a childcare vacuum. This reduces the extent of careful supervision and increases risk of injuries, poor hygiene and nutrition and undermines children's healthy early childhood development (ECD)[4]. In this paper, we present the protocol for a study assessing the demand for, and feasibility of providing day-care for under-5 year olds in poor neighbourhoods in Dhaka, Bangladesh.

Dhaka, the capital city of Bangladesh, has a population of approximately 8.5 million[5] and is one of the most densely populated cities in the world. It is estimated that almost a quarter of the city's inhabitants live in slums[6]. Intra-urban differences are stark, with poor nutrition and high prevalence of infectious diseases resulting in 50% of slum-dwelling children with stunted growth compared to 33% in non-slum areas[7]. Furthermore, urbanisation is changing social and gender norms particularly for the poorest families; in Dhaka slums, twice as many women with an under-5 year old child work outside the home compared to women in the rest of the city[8]. However, these families have few options for childcare and 65% of these children are taken to work by mothers or left with older siblings, particularly older sisters, neighbours or friends [9]. Lack of adequate supervision is a key risk factor for childhood injuries in Bangladesh [10]. The impacts of the limited support for young children can also be seen in educational attainment. Only 65% of children from slum communities attend primary school, compared to 84% outside the slums[11]. Drop-out rates



For peer review only - http://bmjopen.bmj.com/site/about/guidelines.xhtml

are more than six times the national average and higher among girls[12], influenced by the practice of elder sisters providing childcare. Figure 1 presents the culmination of preliminary discussions with ECD providers, academics and policy-makers to explain underlying causes of poor under-5 health and ECD as identified by the project team for this study.

The purpose of this paper is to share the study protocol which aims to develop a sustainable day care for the poor families in urban Bangladesh. This paper should be of interest to researchers' policy makers and practitioners interested in methodologies for mapping existing day-care, demand for day-care, planning and implementation of day-care.

### *The rationale for day-care centres in urban slums*

Evidence from across low-income countries shows that by the time children enter primary school, significant gaps exist in children's development across the social gradient; these gaps widen with time[13]. Adverse exposures and poor development in early childhood has life-long consequences, setting children on a lower trajectory and limiting their future development and opportunities. Across populations, this adversely impacts on a country's social and economic development. Intervening during early childhood is now widely recommended and has the potential to mitigate the negative effects of poverty, promoting more equitable opportunities and better outcomes for education, health, and economic productivity [14, 15]. Interventions to influence a child's development should address four key domains: cognitive development, linguistic development, socio-emotional development, and physical well-being and growth[12, 15]. These domains are interlinked and sequential with progress in one domain acting as a catalyst for development in other domains [10].

A growing body of literature demonstrates that the returns to investments in children's early years are substantial, particularly when compared to equivalent investments made later in life[16] and



For peer review only - http://bmjopen.bmj.com/site/about/guidelines.xhtml

provide a cost-efficient strategy to develop a well-trained and capable workforce, leading to better outcomes for poor households[15] and other broader benefits including improved maternal health, increased female labour participation, raising additional tax revenue, and reducing expenditures on social assistance programmes[17]. The rate of return on investments to ECD interventions depends on the focus, duration of exposure, and quality of programmes being implemented, but have been shown to have benefit-cost ratios as high as 17:1[18].

Health benefits of day-care in low income countries (LIC) beyond ECD have also been identified. A study conducted by Mashreky et al[19] found that community-based day-care for under-5s in rural Bangladesh can reduce risk of all-cause mortality by 44% (95% CI: 20% to 61%), drowning by 82% (95% CI: 42% to 94%) and injuries by 88% (95% CI: 61% to 96%)[9]. While day-care has the potential for significant impact on a wider range of health outcomes, there has been limited research in this area. A Cochrane systematic review[20] assessing the impact of day-care in low-income countries on diarrhoea and respiratory infections found only one study eligible for inclusion. This study found centre-based day-care in Kenya, Uganda and Tanzania/Zanzibar (256 children) had positive effects on the cognitive development of children, but did not report effects on children's psychosocial development, the incidence or prevalence of infectious diseases, parental employment or household income.

A systematic review by Leroy et al.[21] looked more broadly at health, nutrition and cognitive development and found six centre-based studies from Latin America which met the inclusion criteria. The two included studies found large positive effects on child development, as measured by improved school readiness. Due to methodological issues in the included studies, they were unable to draw conclusions regarding impact on child health and nutrition or on the pathways of impact. Both reviews call for further robust research into the impacts of day-care on child health and wider social and educational outcomes.



For peer review only - http://bmjopen.bmj.com/site/about/guidelines.xhtml

In light of this, our study aims to identify the demand for day-care among urban poor communities and test the feasibility of delivering a day-care model that can meet the needs of low-income urban families working long hours whilst being self-sustaining and scaled-up across Dhaka. In the long term, the aim is to address the lack of safe, stimulating and health promoting environments for adequate ECD for 1 to 4 year-old children in disadvantaged urban areas in LICs. We have selected the 1-4 years age group for this intervention as they are at most risk of injuries[22], poor nutrition and poor social and cognitive outcomes[2, 3]; those over 5 are eligible for government pre-school in Bangladesh[23].

# 2 METHODS/DESIGN

*Aims and Objectives*

Our specific objectives are:

In Phase 1:

1. To map and understand day-care models currently being implemented in Dhaka.
2. To understand the extent of demand for day-care, its nature and determinants in slum communities and neighbouring non-slum communities in Dhaka.
3. To identify the most feasible and acceptable method of assessing ECD among under 5 year olds in urban contexts similar to those found in Bangladesh

In Phase 2:

4. To develop, test and refine an integrated model of day-care than can feasibly be delivered in poor urban neighbourhoods in Dhaka.



For peer review only - http://bmjopen.bmj.com/site/about/guidelines.xhtml

In Phase 3:

5. To evaluate the integrated model from the perspective of day-care staff, parents and community members.

6. To assess the feasibility of following-up a potential intervention group at 6 months after base-line measures are taken.

*The Intervention*

The intervention will draw on the WHO/UNICEF evidence-based model of Care for Child Development (CCD) [24, 25]which emphases play and communication activities for care-givers. The materials and experience of other day-care providers in Bangladesh will also be draw on the Centre for Injury Prevention and Research Bangladesh's (CIPRB) 'Anchal' model (shown to be effective in reducing all-cause mortality)[26]. We plan an integrated model which also addresses key areas of public health with children and their parents and care givers; hygiene – (hand washing, and latrine use); promotion of immunisation and access to local primary care services; nutrition through education and provision of healthy meals and snacks; and education on burn and injury prevention. The exact content of the intervention will be determined, tested and adapted using a participatory action research (PAR) approach during the study.

The context of urban slums provides many implementation challenges: the price and availability of land for a large well-equipped child-care centre is high, and ways to generate income for the centre are needed for sustainability; the nature of women's work is different in urban areas compared to rural areas, with longer working hours and considerable travel to garment factories and other work environments; and communities are heterogeneous and transient.

*Study design and methods*

The study has three phases. Phase one will use a sequential mixed methods approach[27] with qualitative data collection building on a household survey to understand day-care and ECD practices and needs in a poor urban neighbourhood in Dhaka. Phase two will use participatory action-research (PAR)[28] with parents and ECD workers to develop a day-care model that can feasibly be delivered. Finally, we will test the feasibility of following-up the families from the poor urban neighbourhood at six months from the baseline measures. The '6SQuID' steps to intervention development have guided development of our approach[23] .

*Study Setting*

For this study, Dhaka ward 56, Kamrangichar, was selected as this area includes both low income "slum' households"[1] and better off non-slum households . It is comprised of three wards, namely wards 55, 56 and 57. Ward 56 of Kamrangichar has been purposively selected as the study area as it exemplifies this mixed slum and non-slum households. The ward includes a mix of residential and small industries. Residential buildings often comprise multi-story dwellings with poorer households living at ground level and wealthier in upper-storey apartments. As informal settlements are cleared within Dhaka, this kind of mixed neighbourhood is increasingly becoming the norm. Thus, assessing feasibility in this type of neighbourhood provides valuable insights for other areas of the city. The mix of wealth categories in the area also means there is the potential to explore whether better-off households would be prepared to pay for day-care and possibly subsidise poorer households. The poorest households in the neighbourhood can consist of 8 family members living in one room and rooms are often clustered together sharing toilets, water sources and cooking facilities. The main occupations, particularly for women in the area are collecting and selling plastic bags and bottles, working as day labourers or as maids in wealthier households.



For peer review only - http://bmjopen.bmj.com/site/about/guidelines.xhtml

A protocol to develop of sustainable day-care for 1-4 year olds in disadvantaged urban communities in Dhaka, Bangladesh

*Study Participants*

Participants for the study are parents and guardians of children under-5 and under 5 children.

*Study Procedures*

*Household Survey (Phase 1): Identifying outcome measures and demand for day-care*

The questionnaire will be pre-tested to identify the most appropriate means of delivery and to check the appropriateness of the questions. Households in Kamrangichar will be surveyed to determine the demand for day-care, current practices and to test the feasibility of collecting data on the outcome measures to be assessed in a future study. These outcomes are ECD, injuries in the last one and six months and body mass index and stunting calculated using height and weight following WHO standards.

In any follow-on study, we will also need to check whether the day-care centre has any impact (positive or negative) on infectious diseases in the community. The questionnaire will therefore collect information on episodes of cough, diarrhoea & vomiting, jaundice or skin rash among under-5 children over the last 1 month.

There are a number of measures of ECD currently available. The survey will help us to identify which is most appropriate to the context in urban Bangladesh, and can be feasibly collected in a larger, future study, to assess the intervention's impact on ECD. In order to identify potential measures of ECD we conducted a review of the literature and held a workshop with international experts on the assessment of ECD. Through this process we identified one measure for under-3s: 1) the Caregiver-Reported Early Development Index (CREDI)[29], and for 3-5 year-olds we identified three measures: 2) Measuring Early Learning Quality and Outcomes (MELQO)[30] ; 3) The Early Human Capability Index (eHCI)[31]; and 4) International Development and Early Learning Assessment (IDELA) [22]. All



For peer review only - http://bmjopen.bmj.com/site/about/guidelines.xhtml

these measures will be collected in the survey on a subset of children and the feasibility and appropriateness of their implementation assessed qualitatively.

The characteristics of these assessment tools are given in Table 1 below. An important difference in the ECD assessments of the over 3 year-olds is whether they are based on the report of the caregiver or through direct assessment. In order to conduct the direct assessments, considerable training of the interviewers is required (around 5 days). This also impacts on the time taken to conduct the interviews. As the IDELA tool requires direct assessment of the child, the interviewers will be trained in its use and how to ensure informed consent is taken from the main caregiver for the direct assessment, in addition to the main questionnaire. The parent or caregiver will be present throughout the assessment and able to break or terminate the assessment at any point based on the child's needs. It may be that during a visit the child does not want to engage in the assessment. If this is the case the assessor will arrange a time to come back.

To assess the feasibility of using each ECD tool in the future we will record the time taken conduct an assessment or caregiver report, and for each question of the measures we will record: 1) its appropriateness to the local context, 2) how easy it is to administer, 3) how well the respondent (either child or caregiver) understands it, 4) the respondents' comfort in answering the question, and 5) its suitability in relation to the child's age. We will also conduct a focus group discussion (FGD) with the data collectors and their supervisors to understand their experiences of using the tools in more depth.

To inform the development of the day-care model to be implemented in phase 2, the household questionnaire will also identify the demand for day-care, current childcare practices, needs and perceptions, including willingness to pay for day-care among both slum and non-slum households.

Sample size: We want to be able to estimate proportional outcomes with 95% confidence intervals of at most ± 10 percentage points. Assuming a design effect due to cluster sampling of 2, this will



For peer review only - http://bmjopen.bmj.com/site/about/guidelines.xhtml

require a sample size of 193 households. Within Kamrangirchar, which contains a roughly equal mix of slum and non-slum households, we will randomly select one mahalla from among the nine mahallas present in which to do the sampling and survey. We will then divide the mahalla into clusters based on the separate "lanes" (main roads) within the mahalla. We will then use local information and exploration to determine the average number of households within each cluster. Then, assuming 10% of households contain at least one under-5 child, we will select the necessary number of clusters to meet our required sample size. We will then visit all households within the cluster to screen them for the presence of under-5 children, and then sample all such households for the survey

As the there is one ECD assessment for those under-3 years and three for those between 3 and 5-years, the following procedures will be followed. In selected households with an under-3 year-old child the CREDI tool will be conducted on one of these children. Selected households with only child aged 3- to 5-years will be randomly split into 3 equal sized groups and children/caregivers allocated to receive either IDELA, MELQO or eHCI assessments.



For peer review only - http://bmjopen.bmj.com/site/about/guidelines.xhtml

A protocol to develop of sustainable day-care for 1-4 year olds in disadvantaged urban communities in Dhaka, Bangladesh

**Table 1: ECD assessments to test during project**

| | **Domains covered** | **Age range** | **Respondents** | **Delivery and Formats available** |
|---|---|---|---|---|
| CREDI Caregiver-Reported Early Development Index (CREDI) | 1) motor development: ability to use fine and gross movements to explore and engage with their environments<br>2) cognitive development, including ability to perceive and discriminate between objects and people, solve problems, communicate needs and desires, understand language, and acquire knowledge<br>3) socioemotional development, including their ability to pay attention, control their impulses, understand their emotions, avoid aggression, anxiety, and distress, and get along with their peers and adults | 0-3 years (36 mths) | Parent/care-giver | Product 1: 20 items for national HHS<br>**Product 2: 60 items (3 domain subscales of 20 items each) good for large scale evaluations of interventions (selected for this study).**<br>Product 3: add-ons relevant to different cultures/regions/governments<br><br>Recommend Product 2, but check if any add-ons for Bangladesh<br><br>In-person interview format, an assessor asks each question out loud to the caregiver, who then responds with a response of Yes, No, or Don't Know. This format is appropriate in contexts in which literacy rates and/or access to the internet are low. |
| IDELA International Development and Early Learning Assessment (SC) | 1) Gross and Fine Motor Development: Hopping on one foot, Copying a shape, Drawing a human figure, Folding Paper<br>2) Emergent Literacy | 42-78 months 3 ½ yrs to 6 ½ yrs | Child | Direct child interview, where a trained assessor sits with a child and follows a scripted protocol for each question, and the assessment of children's approaches to learning is done through assessor observation. After six of the most challenging IDELA items (in many instances novel to children), assessors are asked whether the child was |



For peer review only - http://bmjopen.bmj.com/site/about/guidelines.xhtml

A protocol to develop of sustainable day-care for 1-4 year olds in disadvantaged urban communities in Dhaka, Bangladesh

| | **Domains covered** | **Age range** | **Respondents** | **Delivery and Formats available** |
|---|---|---|---|---|
| | and Language: Print awareness,<br>Expressive vocabulary,<br>Letter identification,<br>Emergent writing,<br>Initial sound discrimination,<br>Listening comprehension<br>3) Emergent numeracy,<br>Measurement and comparison,<br>Classification/Sorting, Number identification,<br>Shape identification,<br>One-to-one correspondence,<br>Simple operations,<br>Simple problem solving<br>4) Socio-emotional Development:<br>Peer relations,<br>Emotional awareness,<br>Empathy,<br>Conflict resolution,<br>Self-awareness<br>Also<br>5) Health and Hygiene and<br>6) 6) Home environment | | Child<br><br>Care-giver | persistent, motivated and attentive in her/his effort to complete the task.<br><br>**Health and Hygiene:** Direct child assessment items are also available and can be added to the assessment to respond to specific health and hygiene interventions that may be occurring within ECD programs. This extended area of assessment is not focused on specific "skills" per se, but instead documents children's knowledge and practices in the following topics: hand washing, teeth brushing, latrine use, healthy food, and use of bed nets.<br><br>**Home Environment**: Care-giver survey of: a safe and nurturing physical environment, opportunities for children to play, explore and learn, and the presence of developmentally appropriate objects, toys and books |
| The Early Human | Social competence<br>Emotional Maturity | 3- 5 years old | Care-giver | Can be completed by parents/care-givers, child care workers, teachers, allied health and other health or ECD |



For peer review only - http://bmjopen.bmj.com/site/about/guidelines.xhtml

A protocol to develop of sustainable day-care for 1-4 year olds in disadvantaged urban communities in Dhaka, Bangladesh

| | **Domains covered** | **Age range** | **Respondents** | **Delivery and Formats available** |
|---|---|---|---|---|
| Capability Index (eHCI) | Language and cognitive<br>approaches to learning<br>Physical health and well-being<br>Perseverance<br>Communication skills | | | practitioner |
| MELQO Measuring Early Learning Quality and Outcomes | - Literacy<br>- Mathematic<br>- Socio-emotional development<br>- Executive function<br>- Physical development<br>- Contextual information | 4-6 years old | Care-giver | Care-giver assessment and direct assessment modules are designed to work together |



For peer review only - http://bmjopen.bmj.com/site/about/guidelines.xhtml

*Qualitative Methods (Phase1)*

We will use qualitative methods to build an in-depth understanding of urban families' day-care practices, requirements and their willingness to pay. The questionnaire will identify those who would, and those who would not, use day-care and the slum/non-slum status of their household[1]. This will enable us to purposively sample ten mothers/primary care-givers for individual interview based on their demand for day-care and whether they live in a slum or non-slum household. Within this purposive sample, we will also include a variety of participants to understand the perspectives of those that may be particularly vulnerable within the community: female headed-households and single mothers; women working outside the home; households with a long-term ill household member; grandparents or other relative or sibling caring for children. The sequential methodology is beneficial in allowing us to conduct this purposive sampling based on participants' questionnaire responses.

We will also interview five carers who are supervising children while their mothers/primary carers work. Once the interviews are completed, then those women who express an interest in using day-care (either during the qualitative interviews or questionnaires) will be invited to attend one of two FGDs. Given the dominant role of husband and senior men in decision-making it will also be important to identify men's and community leaders' attitudes to external day-care. Two FGDs will be held with fathers of under-5 year olds and five semi-structured interviews (SSIs) with community leaders.

*Mapping and understanding current provision of day-care in Dhaka*

The purpose of this component is to map and understand day-care models currently being implemented in Dhaka to inform the design of the model to be tried out as a part of our study. In particular, to identify: 1) the geographical location of existing long day-care centres and low-income areas uncovered; 2) how the different models are being implemented and their key operational



For peer review only - http://bmjopen.bmj.com/site/about/guidelines.xhtml

features; 3) the key features of the well-functioning/ successful and less well-functioning/successful day-care centres; 4 ) the costs of the different models; 5 ) any challenges to implementation and sustainability of the different models.

Phulki, Ministry of Women and Children Affairs (MoWCA) and Manusha Jonno have been selected to exemplify NGO, government and work-based day-care providers in Dhaka. Each organisation will be visited to discuss the model of day-care used in their centres with the programme officer. Running costs will be detailed in a structured Microsoft Excel spreadsheet. During the visit, the researchers will observe and record how activities are done with the children and how engaged the children are; processes for feeding the children; facilities – rooms, equipment, toys/books/pictures, toilet/hand-washing; any interaction seen with parents; any records kept, policies available; any other aspect the indicates how well the centre is running and why. We will also conduct semi-structured interviews with care-giver staff to understand their experiences of looking after the children and what they think are the things that make the day-care centre run well, with happy children and happy parents.

*Develop, test and refine an integrated self-sustaining day-care model (Phase 2)*

The development process will involve a literature review and a series of technical working group (TWG) meetings with participation from national and local government in Dhaka, international and national NGOs involved in ECD, crèche or day-care provision. Following the initial literature review and meetings of this group, a theory of change for the day-care model has been developed (Figure 2). Based on the findings of the questionnaire and the literature review, the TWG will further develop this theory of change and specify the detailed modalities of the day-care model.

CIPRB will implement the model over a six-month period, training staff and developing all necessary materials. We will use PAR[28] to adapt the prototype model so it is appropriate to the urban slum context. The PAR group will consist of day-care staff, local leaders (e.g. local primary school staff) and parents. They will work through the PAR cycles of plan, act, observe and reflect to co-produce



For peer review only - http://bmjopen.bmj.com/site/about/guidelines.xhtml

the day-care model. The research team will facilitate the PAR group and collect, analyse and share any 'observation' data with the group (e.g. attendance, cost or qualitative data on barriers or facilitators). The researchers will document the process through 'learning histories' [28, 32] chronicling the models' adaptation and development.

*To evaluate the integrated model from the perspective of staff, parents and community members (Phase 3)*

Following the six-month implementation, parents, care-givers (10) of children attending the day-care model, purposively sampled from slum and non-slum, will be interviewed to gain their perspective on the potential health and ECD impacts of the intervention, and the mechanisms leading to these changes. The day-care staff will also be interviewed. The above will give us clearly defined components of the intervention, specification of competencies and training programme/materials for staff, specification of costs and fee structure. We will also attempt to follow-up any parents/guardians who stop using the day-care to understand why. Identifying these participants may be challenging.

*Assessing ECD among day-care children and care-giver questionnaire (Phase3)*

Following assessment of feasibility and acceptability of the ECD assessments within the initial questionnaire (demand for day care, household survey), our team will identify which of the ECD assessments is the most appropriate and feasible to implement. This chosen ECD assessment tool will then be used with all new-starters at our model day-care centre, so this could either be the assessment answered by the parent/care giver or the direct assessment (IDELA). The parent/care-giver will also be asked the standardised questions on injuries, hygiene, diet and meals. This will be repeated on the child's last day (as near as possible) at the day-care centre.



For peer review only - http://bmjopen.bmj.com/site/about/guidelines.xhtml

A protocol to develop of sustainable day-care for 1-4 year olds in disadvantaged urban communities in Dhaka, Bangladesh

*To assess feasibility of following-up day-care users and a potential comparator group (Phase 3)*

We will follow up respondents to the initial household questionnaire (demand for day care household survey) six-months later to identify which approach (e.g. home visit, phone call, SMS) will maximise follow-up among this transient group. We will not repeat the full questionnaire and ECD assessment as the main purpose is to identify the proportion of respondent who can be traced for follow-up in a future study.

*Data Analysis*

*Quantitative data:*

For the survey, we will present descriptive statistics on the sampled population's basic characteristics, and report all categorical outcomes as proportions and all continuous outcomes as means, with their associated 95% confidence intervals. We will also use multiple logistic regression to explore the association between key socio-demographic factors including the household's slum/non-slum location and our key outcomes including demand for day-care, willingness to enrol/pay, illness occurrence, injury occurrence and stunting status. These results will be presented as adjusted odds ratios, associated 95% confidence intervals and p-values. The factors included in the models will be determined theoretically, based on the literature and the findings of our qualitative work. All results will account for the clustered design of the survey, using appropriate methods (Taylor series linearisation) to estimate the variance of outcomes.

*Qualitative data:*

As the objective of identifying the dimensions of demand for day-care is clearly specified a priori, a more deductive approach to analysis has been chosen. Using the NVivo 11, we will use Framework Approach[33, 34] which involves five stages of analysis: familiarization with the data, identification

of a thematic framework, indexing (coding raw data), charting and finally interpretation. This structured approach also facilitates analysis by a team that is spread across Bangladesh and the UK, allowing a collaborative approach within each stage of the analysis.

*Patient and Public Involvement*

No patients or public were involved at the development of the protocol. The results of this study will be disseminated to the parents of under-five children at parents' meetings held at the day-care centre.

# 3 Ethics and Dissemination

Ethical approvals for this study were obtained from the School of Medicine Research Ethics Committee at the Faculty of Medicine and Health at the University of Leeds (ref: MREC16-106) and the Bangladesh Medical Research Council (ref: BMRCAIREC/20 I 6-20 I 9 I 250).

Our data management policy will ensure that all data collection, collation, storage, transfer, analysis, archiving and finally destruction is in line with the latest data protection EU legislations, with the UK Data Protection Act 1998, the University of Leeds Data Protection Code of Practice, and local national regulations in Bangladesh. No personal data will be collected from research participants except for names and contact information for the purposes of arranging FGDs and SSIs and following-up survey participants. All information collected through audio-recordings will be anonymised during transcription and before analysis, and participants will be informed of this during the recruitment and consent process.

Utilising appropriate channels to communicate the key findings of research to inform evidence based policy making and practice is an essential aspect of a health services research. We will consistently work with decision makers at the national, regional and the local actors in order to



For peer review only - http://bmjopen.bmj.com/site/about/guidelines.xhtml

embed the study findings into policy and practice. The use of participatory research methods will help ensure that adequate feedback from implementing the intervention is obtained and addressed if required to improve the quality of the model.

More specifically, the results will be disseminated through:

1. Presentations at national, regional and international conferences and publication of articles in peer-reviewed journals with specific emphasis on open access where feasible.

2. Oral presentations at review meetings, conferences and government events at district and national levels in Bangladesh.

3. Developing policy briefs addressed to national and international policymakers and practitioners and designed as short and practical documents.

4. Potential media coverage nationally (e.g., radio television and newspapers) to communicate and promote the research findings creating awareness on the intervention.

5. Developing a dedicated website for the project where the project results will be publicly accessible by national and international decision-makers, practitioners and academics.

# 4 Discussion

Within the context of rapid urbanisation and economic development there is an urgent need for good quality day-care to provide a safe and healthy place for under-5 children. Child day-care presents a holistic solution, allowing women to work whilst knowing their children are safe and provided with ECD opportunities. The potential outcomes of such interventions are multiple as they address health and wider social determinants and economic development.

Our study will provide valuable insights into how to design and deliver day-care that is accessible to poor urban households. Our continual engagement with policy makers and practitioners will help to



For peer review only - http://bmjopen.bmj.com/site/about/guidelines.xhtml

explore options for scale-up of a sustainable model. Few studies have explored current early child-care practices, the need and demand for day-care in Bangladesh or other low and lower-middle-income countries. This study should provide useful insights to those in research and practice who are keen to identify effective and appropriate day-care models.

As described above, there are currently a plethora of tools to assess ECD. There have been recent attempts to synthesise ECD tools, resulting in the development of MELQO [30] This is timely given that Sustainable Development Goal (SDG) Target 4.2, which aims that, by the year 2030, 'all girls and boys have access to quality ECD , care and pre-primary education so that they are ready for primary education'[6]. Our findings will contribute to the knowledge base of the feasibility and acceptability of these tools in an urban low-income context.

A limitation of our study is that, due to the resources available, we are only able to pilot our day-care model in one community. Clearly, insights from different types of urban neighbourhoods would have been valuable in informing the scale up of the day-care model. Similarly, our questionnaire is conducted only one ward of Dhaka. While our sample size is sufficiently powered to provide estimations of demand for day-care, the external validity of the questionnaire to infer to other types of neighbourhoods within Dhaka and other cities in low-income countries is low.

The purpose of this paper is to share the study protocol for developing sustainable day-care centres for 1-4 year olds in Bangladesh. This paper should be of interest to researchers interested in methodologies for assessing procedures for setting up sustainable models of day-care for under-five in urban slums. Policymakers and practitioners may also value a sustainable day-care model characterised by a strong sense of community ownership which is challenging in a mobile population. We plan to collaborate with multiple stakeholders and produce a day-care model based



For peer review only - http://bmjopen.bmj.com/site/about/guidelines.xhtml

on principles of child development with provision of nutritious meals, hygiene and education for children aged 1-4 years.

This intervention has the potential to improve a number of child health and ECD outcomes. During the study, we will identify the most appropriate primary outcome that is important to policy makers, feasible to measure and likely to be sensitive to the effects of the intervention. The outcomes we expect are:

1) Improved ECD: including physical, socio-cognitive, language and learning development, facilitated by inputs from trained childcare workers and their advice to care-givers.

2) Reduced accidents and injuries: being in well-supervised safe day-care will reduce exposure to accidents and injuries. Risk reduction will also be part of advice given to care-givers.

3) Reduced stunting (linear growth retardation): through i) improved nutrition due to provision of healthy meals during day-care and care-giver advice and ii) reduced gastro-intestinal disease due to improved sanitation, provision of clean water and handwashing education for children in day-care.

Financial sustainability is key for longevity and scale-up. This approach will promote integration between poorer and better-off families, potentially facilitating social mobility for slum children. The intervention will draw on the WHO/UNICEF evidence-based model of Care for Child Development [1, 31] which emphasises play and communication activities.

# 5 Collaborations

This project is led by Dr Elsey (UoL) who has led a number of public health intervention development, feasibility and evaluation studies. She brings expertise in public health intervention development, cross-sectional surveys, qualitative methods and PAR. Dr Das (Research Fellow) has a research experience of working in slums in low-income settings in South-East Asia and skills in



For peer review only - http://bmjopen.bmj.com/site/about/guidelines.xhtml

qualitative research. Dr Hicks brings statistical expertise. All have experience of supporting research with partners from low income settings.

CIPRB and ARK Foundation are health research NGOs with impressive portfolios of public health research and strong links to the government and health sector in Bangladesh. Dr Mashreky (CIPRB) has extensive experience of conducting household surveys. Over the last 10 years, he has led the design, implementation and evaluation of the day-care model in rural Bangladesh. CIPRB provide a strong research environment with the infrastructure required to conduct large cross-sectional studies and carefully implement the day-care model, with PAR expertise. Dr Wallace provides additional advice on injuries. Dr Huque and ARK Foundation are long-standing partners with UoL through the DFID-funded COMDIS-HSD consortium. They bring well developed qualitative skills, and strong links with local and national government. Our collaboration includes key players in ECD: Save the Children, Aga Khan Foundation and UNICEF, all of whom attended preliminary workshops in Dhaka. Save the Children will provide in-kind advisory support on ECD and training on the IDELA tool.

# 6 Authors' contributions

HE, MS, RH and HW conceived of the study. MD drafted the manuscript. All authors participated in the study design and contributed to refinement of the paper. All authors have read and approved the final version.

# 7 Acknowledgements

This project has been funded by the MRC Public Health Intervention Development Grant: MR/P017037/1. The funding source had no role in the design and conduct of the study; collection, management, analysis and interpretation of the data; preparation, review and approval of the manuscript; and decision to submit the manuscript for publication.



For peer review only - http://bmjopen.bmj.com/site/about/guidelines.xhtml

# 8 Competing interests

None declared.

# 9 DATA SHARING STATEMENT

Our manuscript describes a study protocol. As such, we cannot elaborate on additional unpublished data.

# 10 FIGURE LEGENDS

Figure 1: Causal pathways perpetuating poor Early Childhood Development among disadvantaged urban 1-4 olds.

Figure 2: Theory of change: Day care in urban poor neighbourhoods.

DRAFT For peer review only

# 11 REFERENCES


1. UNHABITAT, *State of the world's cities 2006/7.* UN-Habitat: London, UK., 2007
2. Walker, S.P., et al., *Inequality in early childhood: risk and protective factors for early child development.* Lancet, 2011. **378**(9799): p. 1325-38.
3. Nair, M.K. and S.R. Radhakrishnan, *Early childhood development in deprived urban settlements.* Indian Pediatr, 2004. **41**(3): p. 227-37.
4. WHO, *Global report on urban health: equitable healthier cities for sustainable development*, 2016: Geneva.
5. World-Population-Review, 2018, World Popluation Review.
6. UNDESA, *World Urbanization Prospects: The 2014 Revision*, 2015, United Nations Department of Economic and Social Affairs/Population Division
7. Angeles, G., et al., *The 2005 census and mapping of slums in Bangladesh: design, select results and application.* International Journal of Health Geographics [Electronic Resource], 2009. **8**: p. 32.
8. Hamadani, J.D., et al., *Use of family care indicators and their relationship with child development in Bangladesh.* Journal of Health, Population & Nutrition, 2010. **28**(1): p. 23-33.
9. Rahman, F., et al., *Cost-Effectiveness of an Injury and Drowning Prevention Program in Bangladesh.* Pediatrics, 2012. **130**(6): p. e1621-e1628.
10. Shonkoff, J.P. and A.S. Garner, *The Lifelong Effects of Early Childhood Adversity and Toxic Stress.* Pediatrics, 2011.
11. Pathey, P., *Multiple Indicator Cluster Survey* 2009.
12. Vegas, E.S., L., *The Promise of Early Childhood Development in Latin America and the Caribbean*. 2010: The World Bank.
13. TheWorldBank., *Investing in Early Childhood Development- reveiw of the World Bank*, 2015.
14. Heckman, J.J., *The Case for Investing in Disadvantaged Young Children*. In Big Ideas for Children: Investing in Our Nation's Future, ed. First Focus. 2008a: Washington, DC: First Focus.
15. Naudeau, S.M., Sebastian; Premand, Patrick; Filmer, Deon *Cognitive Development among Young Children in Low-income countries*. No Small Matter: The Impact of Poverty, Shocks, and Human Capital Investments in Early Childhood Development,, ed. H. Alderman. 2011: World Bank.
16. Heckman, J.J.M.V., *The Productivity Argument for Investing in Young Children.* Review of Agricultural Economics, 2007. **29, number 3**: p. 446-493.
17. Moss, P., G. Dahlberg, and A.Pence. , *Getting beyond the Problem with Quality.* European Early Childhood Education Research Journal 2000. **8 (2)** p. 103-15.
18. Engle, P.L., et al., *Strategies for reducing inequalities and improving developmental outcomes for young children in low-income and middle-income countries.* Lancet, 2011. **378**(9799): p. 1339-53.
19. Mashreky, S.R., et al., *Epidemiology of childhood burn: Yield of largest community based injury survey in Bangladesh.* Burns, 2008. **34**(6): p. 856-862.
20. Brown, T.W., et al., *Centre-based day care for children younger than five years of age in low- and middle-income countries.* Cochrane Database of Systematic Reviews, 2014. **9**: p. CD010543.
21. Leroy, J., P,Gadsden, and M,Guijarro., *The Impact of Daycare Programs On Child Health, Nutrition And Development In Developing Countries: A Systematic Review 3iE.* The International Initiative for Impact Evaluation (3ie). 2011.




For peer review only - http://bmjopen.bmj.com/site/about/guidelines.xhtml

22. Pisani, L., I. Borisova, and A. J. Dowd., *International Development and Early Learning Assessment Technical Working Paper*, 2015, Save the Children Fund.

23. GoB, *Comprehensive Early Childhood Care and Development Policy*, 2013, Ministry of Women and Children Affairs (MOWCA), Government of Bangladesh.

24. *Implementation research toolkit : participants manual.* World Health Organization (WHO), 2014.

25. Jin, X., et al., *"Care for Development" Intervention in Rural China: A Prospective Follow-up Study.* Journal of Developmental & Behavioral Pediatrics, 2007. **28**(3): p. 213-218.

26. Rahman, A., et al. *Mothers' mental health and infant growth: a case-control study from Rawalpindi, Pakistan*. 2004 [cited 2018 25 April]; 1:[21-27].

27. Creswell, J.W., Klassen, A.C., Clark V.L.P., Smith K.C. , *Best Practices for Mixed Methods Research in the Health Sciences* in *The Nature and Design of Mixed Methods Research*2011 OBSSR.

28. Reason, P., Bradbury,H. , *The SAGE Handbook of Action Research Hardcover*, ed. H. Bradbury-Huang. 2015 Sage

29. McCoy DC, S.C., Bellinger DC, Muhihi A, Ashery G, Weary TE, Fawzi W, Fink G. *Development and validation of an early childhood development scale for use in low-resourced settings.* Popul Health Metr 2017 [cited 2018 4 May].

30. UNESCO- UNICEF., B.I., World Bank. *Overview MELQO - Measuring Early Learning Quality and Outcomes*. 2017.

31. Brinkman, S.A., et al., *The role of preschool quality in promoting child development: evidence from rural Indonesia.* European Early Childhood Education Research Journal, 2017. **25**(4): p. 483-505.

32. Bradbury, H. and C. Mainemelis, *Learning History and Organizational Praxis.* Journal of Management Inquiry, 2001. **10**: p. 340.

33. Ritchie J, L.J., *Qualitative research practice: a guide for social science students and researchers.* 2003, London: : Sage.

34. Ritchie J., S., L., *Qualitative data analysis for applied policy research*, in *Analyzing qualitative data*. 1994. p. 173-194.




For peer review only - http://bmjopen.bmj.com/site/about/guidelines.xhtml

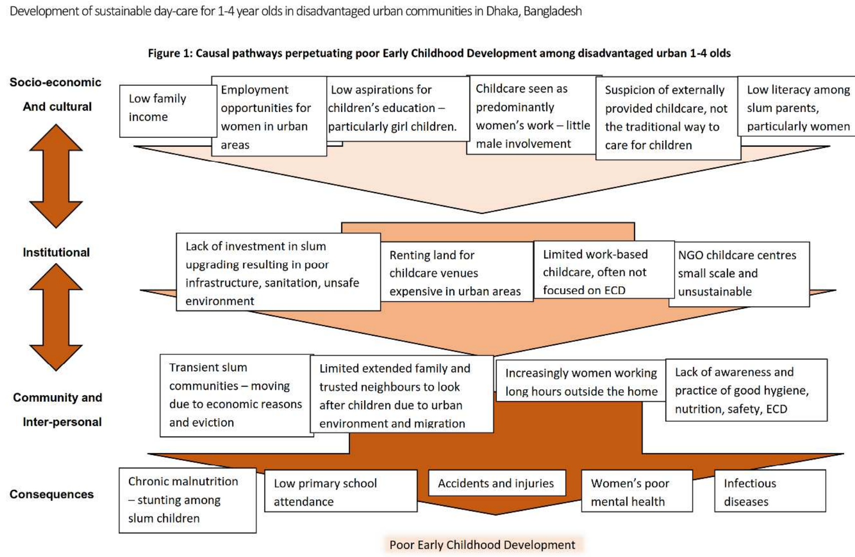


Figure 1: Causal pathways perpetuating poor Early Childhood Development among disadvantaged urban 1-4 olds.

297x209mm (300 x 300 DPI)

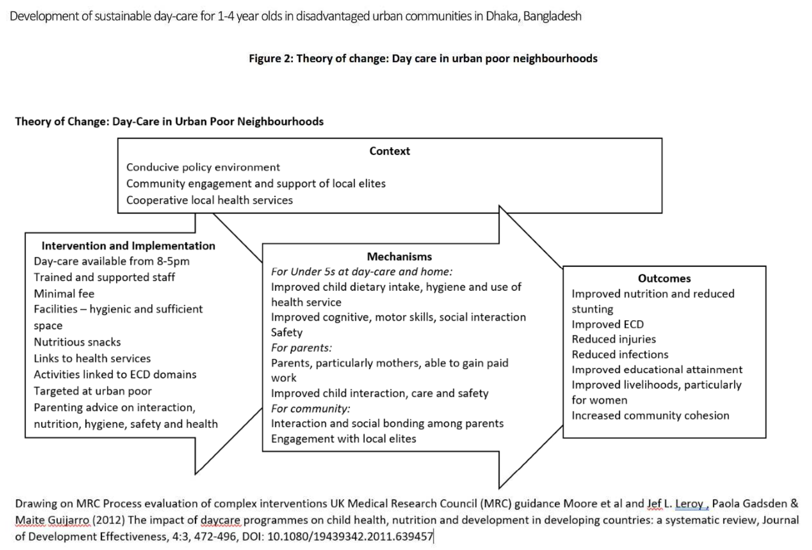


Figure 2: Theory of change: Day care in urban poor neighbourhoods.

297x209mm (300 x 300 DPI)

For peer review only - http://bmjopen.bmj.com/site/about/guidelines.xhtml

# A protocol to develop sustainable day-care for 1-4 year olds in disadvantaged urban communities in Dhaka, Bangladesh



| | |
|---|---|
| Journal: | *BMJ Open* |
| Manuscript ID | bmjopen-2018-024101.R1 |
| Article Type: | Protocol |
| Date Submitted by the Author: | 11-Jun-2018 |



| | |
|---|---|
| Complete List of Authors: | Das, Mahua; Leeds University, Leeds Institute of Health Sciences<br>Elsey, Helen; University of Leeds, Nuffield Centre for International Health and Development<br>Shawon , R.A; Centre for Injury Prevention and Research Bangladesh ,<br>Hicks, Joseph; University of Leeds, Nuffield Centre for International Health and Development, Leeds Institute of Health Sciences<br>Ferdoush, J; Centre for Injury Prevention and Research Bangladesh<br>Huque, Rumana; University of Dhaka, Economics<br>Fieroze, Fariza; ARK Foundation<br>Nasreen, Shammi; ARK Foundation,<br>Wallace, Hilary; The University of Notre Dame Australia, School of Medicine,<br>Mashreky, Saidur; Centre for Injury Prevention and Research Bangladesh, |



| | |
|---|---|
| <b>Primary Subject Heading</b>: | Global health |
| Secondary Subject Heading: | Health services research |
| Keywords: | Protocols & guidelines < HEALTH SERVICES ADMINISTRATION & MANAGEMENT, Child protection < PAEDIATRICS, Community child health < PAEDIATRICS, PUBLIC HEALTH, QUALITATIVE RESEARCH |


SCHOLARONE™
Manuscripts

# A protocol to develop sustainable day-care for 1-4 year olds in disadvantaged urban communities in Dhaka, Bangladesh

Authors:

1. **Das, M.** Nuffield Institute for International Health and Development, University of Leeds, m.das@leeds.ac.uk ; Phone number: 0044-7809601932
2. **Elsey, H**. Nuffield Institute for International Health and Development, University of Leeds, h.elsey@leeds.ac.uk.
3. **Shawon, RA.** Centre for Injury Prevention and Research Bangladesh (CIPRB) riasha47@gmail.com
4. **Hicks, J**. Nuffield Institute for International Health and Development, University of Leeds, J.P.Hicks@leeds.ac.uk
5. **Ferdoush, J.** Centre for Injury Prevention and Research Bangladesh (CIPRB), junnatul.ferdoush@gmail.com
6. **Huque, R**. ARK Foundation, rumanah14@yahoo.com
7. **Fieroze, F**. ARK Foundation farizafieroze@yahoo.com
8. **Nasreen, S**. ARK Foundation, shammibadrul@yahoo.com
9. **Wallace, H**. The University of Notre Dame Australia, School of Medicine, Fremantle, hilary.wallace@nd.edu.au
10. **Mashreky, SR.** Centre for Injury Prevention and Research Bangladesh (CIPRB) mashreky@ciprb.org

**Word count**- 5,282 words excluding the abstract, tables, figures and references.



For peer review only - http://bmjopen.bmj.com/site/about/guidelines.xhtml

A protocol to develop of sustainable day-care for 1-4 year olds in disadvantaged urban communities in Dhaka, Bangladesh

**Abstract:**

**Introduction**: Lack of safe, stimulating and health-promoting environments for children under-5 hinders their physical, social and cognitive development, known as Early Childhood Development (ECD). Improving ECD not only impacts on children, but can improve educational attainment for girls, who often care for younger siblings, and employment prospects for mothers. Developing and evaluating the impacts of ECD programmes within childcare needs to assess a range of social, health, educational and economic impacts, including women's empowerment.

Children living in slums are at high risk of poor early development and holistic, sustainable interventions are needed to address ECD in these contexts. This study will be undertaken in Dhaka, Bangladesh, a city where over 8.5 million inhabitants live in slums. In collaboration with government, NGOs and communities, we are developing and testing a sustainable day-care model for low-income communities in Dhaka.

**Methodology and Analysis**:

A sequential mixed methods approach is being used in the study, with qualitative work exploring quantitative findings. Two hundred households with children under-5 will be surveyed to determine day-care needs and to assess ECD (parent reported and direct assessment). The feasibility of four ECD measuring tools Caregiver-Reported Early Development Index , Measuring Early Learning Quality and Outcomes, The Early Human Capability Index and International Development and Early Learning Assessment will be assessed quantitative and qualitatively. Qualitative methods will help understand demand and perceptions of day-care while mothers work. Participatory action research will be used to develop a locally appropriate and potentially sustainable model of day-care for under 5 children. A ward in the south of Dhaka has been selected for the study as this typifies communities with slum and non-slum households living next to each other, allowing us to explore potential for better-off household to subsidise day-care for poorer households.

**Ethics and dissemination**:

Findings will be published and inform decision makers at the national, regional and the local actors in order to embed the study into the policy and practice on childcare and ECD. University of Leeds Ethical Review Board approved: (ref: MREC16-106) and the Bangladesh Medical Research Council (ref: BMRCAIREC/20 I 6-20 I 9 I 250

**Keywords:** Urban slum, Day-care, Under-5 children, Early childhood development, Bangladesh, Low income countries



For peer review only - http://bmjopen.bmj.com/site/about/guidelines.xhtml

A protocol to develop of sustainable day-care for 1-4 year olds in disadvantaged urban communities in Dhaka, Bangladesh

**Strengths and weakness of the study**

**Strengths**:

1. Our participatory action research will provide valuable insights into how to design and deliver day-care that is accessible to poor urban households.
2. Our survey will fill a gap in the evidence by providing information on the demand for day-care among slum and non-slum households.
3. Continual engagement with policy makers and practitioners will help to explore options for scale-up of a sustainable model.

**Weaknesses**:

1. Due to the available resources we are only able to develop the day-care model in one community, potentially limiting the identification of delivery issues that might occur in other urban neighbourhoods.
2. Our questionnaire is only conducted in one ward of Dhaka. While our sample size is sufficiently powered to provide estimations of demand for day-care, the external validity of the questionnaire to make inferences to other types of neighbourhoods within Dhaka and other low-income cities is limited.



For peer review only - http://bmjopen.bmj.com/site/about/guidelines.xhtml

A protocol to  develop of sustainable day-care for 1-4 year olds in disadvantaged urban communities in Dhaka, Bangladesh

# 1 Background

Rapid urbanization, with over 60 per cent of the world's population is expected to live in cities by 2030 and nearly 70 per cent by 2050, is a major global concern and has led to a sustainable development goal focused specifically on sustainable cities [1]. Much of this growth is seen in low income countries where an increasing number of the urban population live in slum conditions[1] Such conditions increase exposure to communicable diseases and risk factors for non-communicable diseases increasing the risk of poor health, social and cognitive outcomes during childhood and later in life [2, 3]. With slum-dwelling families, particularly women, working long hours and limited availability of extended family, slum communities face a childcare vacuum. This reduces the extent of careful supervision and increases risk of injuries, poor hygiene and nutrition and undermines children's healthy early childhood development (ECD)[4]. In this paper, we present the protocol for a study assessing the demand for, and feasibility of providing day-care for under-5 year olds in poor neighbourhoods in Dhaka, Bangladesh.

Dhaka, the capital city of Bangladesh, has a population of approximately 8.5 million[5] and is one of the most densely populated cities in the world. It is estimated that almost a quarter of the city's inhabitants live in slums[6]. Intra-urban differences are stark, with poor nutrition and high prevalence of infectious diseases resulting in 50% of slum-dwelling children with stunted growth compared to 33% in non-slum areas[7]. Furthermore, urbanisation is changing social and gender norms particularly for the poorest families; in Dhaka slums, twice as many women with an under-5 year old child work outside the home compared to women in the rest of the city[8]. However, these families have few options for childcare and 65% of these children are taken to work by mothers or left with older siblings, particularly older sisters, neighbours or friends [9]. Lack of adequate supervision is a key risk factor for childhood injuries in Bangladesh [10]. The impacts of the limited support for young children can also be seen in educational attainment. Only 65% of children from slum communities attend primary school, compared to 84% outside the slums[11]. Drop-out rates



For peer review only - http://bmjopen.bmj.com/site/about/guidelines.xhtml

are more than six times the national average and higher among girls[12], influenced by the practice of elder sisters providing childcare. Figure 1 presents the culmination of preliminary discussions with ECD providers, academics and policy-makers to explain underlying causes of poor under-5 health and ECD as identified by the project team for this study.

The purpose of this paper is to share the study protocol which aims to develop a sustainable day care for the poor families in urban Bangladesh. This paper should be of interest to researchers' policy makers and practitioners interested in methodologies for mapping existing day-care, demand for day-care, planning and implementation of day-care.

### *The rationale for day-care centres in urban slums*

Evidence from across low-income countries shows that by the time children enter primary school, significant gaps exist in children's development across the social gradient; these gaps widen with time[13]. Adverse exposures and poor development in early childhood has life-long consequences, setting children on a lower trajectory and limiting their future development and opportunities. Across populations, this adversely impacts on a country's social and economic development. Intervening during early childhood is now widely recommended and has the potential to mitigate the negative effects of poverty, promoting more equitable opportunities and better outcomes for education, health, and economic productivity [14, 15]. Interventions to influence a child's development should address four key domains: cognitive development, linguistic development, socio-emotional development, and physical well-being and growth[12, 15]. These domains are interlinked and sequential with progress in one domain acting as a catalyst for development in other domains [10].

A growing body of literature demonstrates that the returns to investments in children's early years are substantial, particularly when compared to equivalent investments made later in life[16] and



For peer review only - http://bmjopen.bmj.com/site/about/guidelines.xhtml

provide a cost-efficient strategy to develop a well-trained and capable workforce, leading to better outcomes for poor households[15] and other broader benefits including improved maternal health, increased female labour participation, raising additional tax revenue, and reducing expenditures on social assistance programmes[17]. The rate of return on investments to ECD interventions depends on the focus, duration of exposure, and quality of programmes being implemented, but have been shown to have benefit-cost ratios as high as 17:1[18].

Health benefits of day-care in low income countries (LIC) beyond ECD have also been identified. A study conducted by Mashreky et al[19] found that community-based day-care for under-5s in rural Bangladesh can reduce risk of all-cause mortality by 44% (95% CI: 20% to 61%), drowning by 82% (95% CI: 42% to 94%) and injuries by 88% (95% CI: 61% to 96%)[9]. While day-care has the potential for significant impact on a wider range of health outcomes, there has been limited research in this area. A Cochrane systematic review[20] assessing the impact of day-care in low-income countries on diarrhoea and respiratory infections found only one study eligible for inclusion. This study found centre-based day-care in Kenya, Uganda and Tanzania/Zanzibar (256 children) had positive effects on the cognitive development of children, but did not report effects on children's psychosocial development, the incidence or prevalence of infectious diseases, parental employment or household income.

A systematic review by Leroy et al.[21] looked more broadly at health, nutrition and cognitive development and found six centre-based studies from Latin America which met the inclusion criteria. The two included studies found large positive effects on child development, as measured by improved school readiness. Due to methodological issues in the included studies, they were unable to draw conclusions regarding impact on child health and nutrition or on the pathways of impact. Both reviews call for further robust research into the impacts of day-care on child health and wider social and educational outcomes.



For peer review only - http://bmjopen.bmj.com/site/about/guidelines.xhtml

In light of this, our study aims to identify the demand for day-care among urban poor communities and test the feasibility of delivering a day-care model that can meet the needs of low-income urban families working long hours whilst being self-sustaining and scaled-up across Dhaka. In the long term, the aim is to address the lack of safe, stimulating and health promoting environments for adequate ECD for 1 to 4 year-old children in disadvantaged urban areas in LICs. We have selected the 1-4 years age group for this intervention as they are at most risk of injuries[22], poor nutrition and poor social and cognitive outcomes[2, 3]; those over 5 are eligible for government pre-school in Bangladesh[23].

# 2 METHODS/DESIGN

*Aims and Objectives*

Our specific objectives are:

In Phase 1:

1. To map and understand day-care models currently being implemented in Dhaka.
2. To understand the extent of demand for day-care, its nature and determinants in slum communities and neighbouring non-slum communities in Dhaka.
3. To identify the most feasible and acceptable method of assessing ECD among under 5 year olds in urban contexts similar to those found in Bangladesh

In Phase 2:

4. To develop, test and refine an integrated model of day-care than can feasibly be delivered in poor urban neighbourhoods in Dhaka.



For peer review only - http://bmjopen.bmj.com/site/about/guidelines.xhtml

In Phase 3:

5. To evaluate the integrated model from the perspective of day-care staff, parents and community members.

6. To assess the feasibility of following-up a potential intervention group at 6 months after base-line measures are taken.

*The Intervention*

The intervention will draw on the WHO/UNICEF evidence-based model of Care for Child Development (CCD) [24, 25]which emphases play and communication activities for care-givers. The materials and experience of other day-care providers in Bangladesh will also be draw on the Centre for Injury Prevention and Research Bangladesh's (CIPRB) 'Anchal' model (shown to be effective in reducing all-cause mortality)[26]. We plan an integrated model which also addresses key areas of public health with children and their parents and care givers; hygiene – (hand washing, and latrine use); promotion of immunisation and access to local primary care services; nutrition through education and provision of healthy meals and snacks; and education on burn and injury prevention. The exact content of the intervention will be determined, tested and adapted using a participatory action research (PAR) approach during the study.

The context of urban slums provides many implementation challenges: the price and availability of land for a large well-equipped child-care centre is high, and ways to generate income for the centre are needed for sustainability; the nature of women's work is different in urban areas compared to rural areas, with longer working hours and considerable travel to garment factories and other work environments; and communities are heterogeneous and transient.



For peer review only - http://bmjopen.bmj.com/site/about/guidelines.xhtml

A protocol to develop of sustainable day-care for 1-4 year olds in disadvantaged urban communities in Dhaka, Bangladesh

*Study design and methods*

The study has three phases. Phase one will use a sequential mixed methods approach[27] with qualitative data collection building on a household survey to understand day-care and ECD practices and needs in a poor urban neighbourhood in Dhaka. Phase two will use participatory action-research (PAR)[28] with parents and ECD workers to develop a day-care model that can feasibly be delivered. Finally, we will test the feasibility of following-up the families from the poor urban neighbourhood at six months from the baseline measures. The '6SQuID' steps to intervention development have guided development of our approach[23] .

*Study Setting*

For this study, Dhaka ward 56, Kamrangichar, was selected as this area includes both low income "slum' households"[1] and better off non-slum households . It is comprised of three wards, namely wards 55, 56 and 57. Ward 56 of Kamrangichar has been purposively selected as the study area as it exemplifies this mixed slum and non-slum households. The ward includes a mix of residential and small industries. Residential buildings often comprise multi-story dwellings with poorer households living at ground level and wealthier in upper-storey apartments. As informal settlements are cleared within Dhaka, this kind of mixed neighbourhood is increasingly becoming the norm. Thus, assessing feasibility in this type of neighbourhood provides valuable insights for other areas of the city. The mix of wealth categories in the area also means there is the potential to explore whether better-off households would be prepared to pay for day-care and possibly subsidise poorer households. The poorest households in the neighbourhood can consist of 8 family members living in one room and rooms are often clustered together sharing toilets, water sources and cooking facilities. The main occupations, particularly for women in the area are collecting and selling plastic bags and bottles, working as day labourers or as maids in wealthier households.



For peer review only - http://bmjopen.bmj.com/site/about/guidelines.xhtml

*Study Participants*

Participants for the study are parents and guardians of children under-5 and under 5 children.

*Study Procedures*

*Household Survey (Phase 1): Identifying outcome measures and demand for day-care*

The questionnaire will be pre-tested to identify the most appropriate means of delivery and to check the appropriateness of the questions. Households in Kamrangichar will be surveyed to determine the demand for day-care, current practices and to test the feasibility of collecting data on the outcome measures to be assessed in a future study. These outcomes are ECD, injuries in the last one and six months and body mass index and stunting calculated using height and weight following WHO standards.

In any follow-on study, we will also need to check whether the day-care centre has any impact (positive or negative) on infectious diseases in the community. The questionnaire will therefore collect information on episodes of cough, diarrhoea & vomiting, jaundice or skin rash among under-5 children over the last 1 month.

There are a number of measures of ECD currently available. The survey will help us to identify which is most appropriate to the context in urban Bangladesh, and can be feasibly collected in a larger, future study, to assess the intervention's impact on ECD. In order to identify potential measures of ECD we conducted a review of the literature and held a workshop with international experts on the assessment of ECD. Through this process we identified one measure for under-3s: 1) the Caregiver-Reported Early Development Index (CREDI)[29], and for 3-5 year-olds we identified three measures: 2) Measuring Early Learning Quality and Outcomes (MELQO)[30] ; 3) The Early Human Capability Index (eHCI)[31]; and 4) International Development and Early Learning Assessment (IDELA) [22]. All



For peer review only - http://bmjopen.bmj.com/site/about/guidelines.xhtml

these measures will be collected in the survey on a subset of children and the feasibility and appropriateness of their implementation assessed qualitatively.

The characteristics of these assessment tools are given in Table 1 below. An important difference in the ECD assessments of the over 3 year-olds is whether they are based on the report of the caregiver or through direct assessment. In order to conduct the direct assessments, considerable training of the interviewers is required (around 5 days). This also impacts on the time taken to conduct the interviews. As the IDELA tool requires direct assessment of the child, the interviewers will be trained in its use and how to ensure informed consent is taken from the main caregiver for the direct assessment, in addition to the main questionnaire. The parent or caregiver will be present throughout the assessment and able to break or terminate the assessment at any point based on the child's needs. It may be that during a visit the child does not want to engage in the assessment. If this is the case the assessor will arrange a time to come back.

To assess the feasibility of using each ECD tool in the future we will record the time taken conduct an assessment or caregiver report, and for each question of the measures we will record: 1) its appropriateness to the local context, 2) how easy it is to administer, 3) how well the respondent (either child or caregiver) understands it, 4) the respondents' comfort in answering the question, and 5) its suitability in relation to the child's age. We will also conduct a focus group discussion (FGD) with the data collectors and their supervisors to understand their experiences of using the tools in more depth.

To inform the development of the day-care model to be implemented in phase 2, the household questionnaire will also identify the demand for day-care, current childcare practices, needs and perceptions, including willingness to pay for day-care among both slum and non-slum households.

Sample size: We want to be able to estimate proportional outcomes with 95% confidence intervals of at most ± 10 percentage points. Assuming a design effect due to cluster sampling of 2, this will



For peer review only - http://bmjopen.bmj.com/site/about/guidelines.xhtml

require a sample size of 193 households. Within Kamrangirchar, which contains a roughly equal mix of slum and non-slum households, we will randomly select one mahalla from among the nine mahallas present in which to do the sampling and survey. We will then divide the mahalla into clusters based on the separate "lanes" (main roads) within the mahalla. We will then use local information and exploration to determine the average number of households within each cluster. Then, assuming 10% of households contain at least one under-5 child, we will select the necessary number of clusters to meet our required sample size. We will then visit all households within the cluster to screen them for the presence of under-5 children, and then sample all such households for the survey

As the there is one ECD assessment for those under-3 years and three for those between 3 and 5-years, the following procedures will be followed. In selected households with an under-3 year-old child the CREDI tool will be conducted on one of these children. Selected households with only child aged 3- to 5-years will be randomly split into 3 equal sized groups and children/caregivers allocated to receive either IDELA, MELQO or eHCI assessments.



For peer review only - http://bmjopen.bmj.com/site/about/guidelines.xhtml

A protocol to develop of sustainable day-care for 1-4 year olds in disadvantaged urban communities in Dhaka, Bangladesh

Table 1: ECD assessments to test during project

| | Domains covered | Age range | Respondents | Delivery and Formats available |
|---|---|---|---|---|
| CREDI Caregiver-Reported Early Development Index (CREDI) | 1) motor development: ability to use fine and gross movements to explore and engage with their environments<br>2) cognitive development, including ability to perceive and discriminate between objects and people, solve problems, communicate needs and desires, understand language, and acquire knowledge<br>3) socioemotional development, including their ability to pay attention, control their impulses, understand their emotions, avoid aggression, anxiety, and distress, and get along with their peers and adults | 0-3 years (36 mths) | Parent/care-giver | Product 1: 20 items for national HHS<br>**Product 2: 60 items (3 domain subscales of 20 items each) good for large scale evaluations of interventions (selected for this study).**<br>Product 3: add-ons relevant to different cultures/regions/governments<br><br>Recommend Product 2, but check if any add-ons for Bangladesh<br><br>In-person interview format, an assessor asks each question out loud to the caregiver, who then responds with a response of Yes, No, or Don't Know. This format is appropriate in contexts in which literacy rates and/or access to the internet are low. |
| IDELA International Development and Early Learning Assessment (SC) | 1) Gross and Fine Motor Development: Hopping on one foot, Copying a shape, Drawing a human figure, Folding Paper<br>2) Emergent Literacy | 42-78 months 3 ½ yrs to 6 ½ yrs | Child | Direct child interview, where a trained assessor sits with a child and follows a scripted protocol for each question, and the assessment of children's approaches to learning is done through assessor observation. After six of the most challenging IDELA items (in many instances novel to children), assessors are asked whether the child was |

DRAFT For peer review only

A protocol to develop of sustainable day-care for 1-4 year olds in disadvantaged urban communities in Dhaka, Bangladesh

| | **Domains covered** | **Age range** | **Respondents** | **Delivery and Formats available** |
|---|---|---|---|---|
| | and Language: Print awareness,<br>Expressive vocabulary,<br>Letter identification,<br>Emergent writing,<br>Initial sound discrimination,<br>Listening comprehension<br>3) Emergent numeracy,<br>Measurement and comparison,<br>Classification/Sorting, Number identification,<br>Shape identification,<br>One-to-one correspondence,<br>Simple operations,<br>Simple problem solving<br>4) Socio-emotional Development:<br>Peer relations,<br>Emotional awareness,<br>Empathy,<br>Conflict resolution,<br>Self-awareness<br>Also<br>5) Health and Hygiene and<br>6) 6) Home environment | | Child<br><br>Care-giver | persistent, motivated and attentive in her/his effort to complete the task.<br><br>**Health and Hygiene:** Direct child assessment items are also available and can be added to the assessment to respond to specific health and hygiene interventions that may be occurring within ECD programs. This extended area of assessment is not focused on specific "skills" per se, but instead documents children's knowledge and practices in the following topics: hand washing, teeth brushing, latrine use, healthy food, and use of bed nets.<br><br>**Home Environment**: Care-giver survey of: a safe and nurturing physical environment, opportunities for children to play, explore and learn, and the presence of developmentally appropriate objects, toys and books |
| The Early Human | Social competence<br>Emotional Maturity | 3- 5 years old | Care-giver | Can be completed by parents/care-givers, child care workers, teachers, allied health and other health or ECD |



For peer review only - http://bmjopen.bmj.com/site/about/guidelines.xhtml

A protocol to develop of sustainable day-care for 1-4 year olds in disadvantaged urban communities in Dhaka, Bangladesh

| | **Domains covered** | **Age range** | **Respondents** | **Delivery and Formats available** |
|---|---|---|---|---|
| Capability Index (eHCI) | Language and cognitive approaches to learning<br>Physical health and well-being<br>Perseverance<br>Communication skills | | | practitioner |
| MELQO Measuring Early Learning Quality and Outcomes | - Literacy<br>- Mathematic<br>- Socio-emotional development<br>- Executive function<br>- Physical development<br>- Contextual information | 4-6 years old | Care-giver | Care-giver assessment and direct assessment modules are designed to work together |

*Qualitative Methods (Phase1)*

We will use qualitative methods to build an in-depth understanding of urban families' day-care practices, requirements and their willingness to pay. The questionnaire will identify those who would, and those who would not, use day-care and the slum/non-slum status of their household[1]. This will enable us to purposively sample ten mothers/primary care-givers for individual interview based on their demand for day-care and whether they live in a slum or non-slum household. Within this purposive sample, we will also include a variety of participants to understand the perspectives of those that may be particularly vulnerable within the community: female headed-households and single mothers; women working outside the home; households with a long-term ill household member; grandparents or other relative or sibling caring for children. The sequential methodology is beneficial in allowing us to conduct this purposive sampling based on participants' questionnaire responses.

We will also interview five carers who are supervising children while their mothers/primary carers work. Once the interviews are completed, then those women who express an interest in using day-care (either during the qualitative interviews or questionnaires) will be invited to attend one of two FGDs. Given the dominant role of husband and senior men in decision-making it will also be important to identify men's and community leaders' attitudes to external day-care. Two FGDs will be held with fathers of under-5 year olds and five semi-structured interviews (SSIs) with community leaders.

*Mapping and understanding current provision of day-care in Dhaka*

The purpose of this component is to map and understand day-care models currently being implemented in Dhaka to inform the design of the model to be tried out as a part of our study. In particular, to identify: 1) the geographical location of existing long day-care centres and low-income areas uncovered; 2) how the different models are being implemented and their key operational



For peer review only - http://bmjopen.bmj.com/site/about/guidelines.xhtml

features; 3) the key features of the well-functioning/ successful and less well-functioning/successful day-care centres; 4 ) the costs of the different models; 5 ) any challenges to implementation and sustainability of the different models.

Phulki, Ministry of Women and Children Affairs (MoWCA) and Manusha Jonno have been selected to exemplify NGO, government and work-based day-care providers in Dhaka. Each organisation will be visited to discuss the model of day-care used in their centres with the programme officer. Running costs will be detailed in a structured Microsoft Excel spreadsheet. During the visit, the researchers will observe and record how activities are done with the children and how engaged the children are; processes for feeding the children; facilities – rooms, equipment, toys/books/pictures, toilet/hand-washing; any interaction seen with parents; any records kept, policies available; any other aspect the indicates how well the centre is running and why. We will also conduct semi-structured interviews with care-giver staff to understand their experiences of looking after the children and what they think are the things that make the day-care centre run well, with happy children and happy parents.

*Develop, test and refine an integrated self-sustaining day-care model (Phase 2)*

The development process will involve a literature review and a series of technical working group (TWG) meetings with participation from national and local government in Dhaka, international and national NGOs involved in ECD, crèche or day-care provision. Following the initial literature review and meetings of this group, a theory of change for the day-care model has been developed (Figure 2). Based on the findings of the questionnaire and the literature review, the TWG will further develop this theory of change and specify the detailed modalities of the day-care model.

CIPRB will implement the model over a six-month period, training staff and developing all necessary materials. We will use PAR[28] to adapt the prototype model so it is appropriate to the urban slum context. The PAR group will consist of day-care staff, local leaders (e.g. local primary school staff) and parents. They will work through the PAR cycles of plan, act, observe and reflect to co-produce



For peer review only - http://bmjopen.bmj.com/site/about/guidelines.xhtml

the day-care model. The research team will facilitate the PAR group and collect, analyse and share any 'observation' data with the group (e.g. attendance, cost or qualitative data on barriers or facilitators). The researchers will document the process through 'learning histories' [28, 32] chronicling the models' adaptation and development.

*To evaluate the integrated model from the perspective of staff, parents and community members (Phase 3)*

Following the six-month implementation, parents, care-givers (10) of children attending the day-care model, purposively sampled from slum and non-slum, will be interviewed to gain their perspective on the potential health and ECD impacts of the intervention, and the mechanisms leading to these changes. The day-care staff will also be interviewed. The above will give us clearly defined components of the intervention, specification of competencies and training programme/materials for staff, specification of costs and fee structure. We will also attempt to follow-up any parents/guardians who stop using the day-care to understand why. Identifying these participants may be challenging.

*Assessing ECD among day-care children and care-giver questionnaire (Phase3)*

Following assessment of feasibility and acceptability of the ECD assessments within the initial questionnaire (demand for day care, household survey), our team will identify which of the ECD assessments is the most appropriate and feasible to implement. This chosen ECD assessment tool will then be used with all new-starters at our model day-care centre, so this could either be the assessment answered by the parent/care giver or the direct assessment (IDELA). The parent/care-giver will also be asked the standardised questions on injuries, hygiene, diet and meals. This will be repeated on the child's last day (as near as possible) at the day-care centre.



For peer review only - http://bmjopen.bmj.com/site/about/guidelines.xhtml

*To assess feasibility of following-up day-care users and a potential comparator group (Phase 3)*

We will follow up respondents to the initial household questionnaire (demand for day care household survey) six-months later to identify which approach (e.g. home visit, phone call, SMS) will maximise follow-up among this transient group. We will not repeat the full questionnaire and ECD assessment as the main purpose is to identify the proportion of respondent who can be traced for follow-up in a future study.

*Data Analysis*

*Quantitative data:*

For the survey, we will present descriptive statistics on the sampled population's basic characteristics, and report all categorical outcomes as proportions and all continuous outcomes as means, with their associated 95% confidence intervals. We will also use multiple logistic regression to explore the association between key socio-demographic factors including the household's slum/non-slum location and our key outcomes including demand for day-care, willingness to enrol/pay, illness occurrence, injury occurrence and stunting status. These results will be presented as adjusted odds ratios, associated 95% confidence intervals and p-values. The factors included in the models will be determined theoretically, based on the literature and the findings of our qualitative work. All results will account for the clustered design of the survey, using appropriate methods (Taylor series linearisation) to estimate the variance of outcomes.

*Qualitative data:*

As the objective of identifying the dimensions of demand for day-care is clearly specified a priori, a more deductive approach to analysis has been chosen. Using the NVivo 11, we will use Framework Approach[33, 34] which involves five stages of analysis: familiarization with the data, identification



For peer review only - http://bmjopen.bmj.com/site/about/guidelines.xhtml

of a thematic framework, indexing (coding raw data), charting and finally interpretation. This structured approach also facilitates analysis by a team that is spread across Bangladesh and the UK, allowing a collaborative approach within each stage of the analysis.

*Patient and Public Involvement*

No patients or public were involved at the development of the protocol. The results of this study will be disseminated to the parents of under-five children at parents' meetings held at the day-care centre.

# 3 Ethics and Dissemination

Ethical approvals for this study were obtained from the School of Medicine Research Ethics Committee at the Faculty of Medicine and Health at the University of Leeds (ref: MREC16-106) and the Bangladesh Medical Research Council (ref: BMRCAIREC/20 I 6-20 I 9 I 250).

Our data management policy will ensure that all data collection, collation, storage, transfer, analysis, archiving and finally destruction is in line with the latest data protection EU legislations, with the UK Data Protection Act 1998, the University of Leeds Data Protection Code of Practice, and local national regulations in Bangladesh. No personal data will be collected from research participants except for names and contact information for the purposes of arranging FGDs and SSIs and following-up survey participants. All information collected through audio-recordings will be anonymised during transcription and before analysis, and participants will be informed of this during the recruitment and consent process.

Utilising appropriate channels to communicate the key findings of research to inform evidence based policy making and practice is an essential aspect of a health services research. We will consistently work with decision makers at the national, regional and the local actors in order to



For peer review only - http://bmjopen.bmj.com/site/about/guidelines.xhtml

embed the study findings into policy and practice. The use of participatory research methods will help ensure that adequate feedback from implementing the intervention is obtained and addressed if required to improve the quality of the model.

More specifically, the results will be disseminated through:

1. Presentations at national, regional and international conferences and publication of articles in peer-reviewed journals with specific emphasis on open access where feasible.

2. Oral presentations at review meetings, conferences and government events at district and national levels in Bangladesh.

3. Developing policy briefs addressed to national and international policymakers and practitioners and designed as short and practical documents.

4. Potential media coverage nationally (e.g., radio television and newspapers) to communicate and promote the research findings creating awareness on the intervention.

5. Developing a dedicated website for the project where the project results will be publicly accessible by national and international decision-makers, practitioners and academics.

# 4 Discussion

Within the context of rapid urbanisation and economic development there is an urgent need for good quality day-care to provide a safe and healthy place for under-5 children. Child day-care presents a holistic solution, allowing women to work whilst knowing their children are safe and provided with ECD opportunities. The potential outcomes of such interventions are multiple as they address health and wider social determinants and economic development.

Our study will provide valuable insights into how to design and deliver day-care that is accessible to poor urban households. Our continual engagement with policy makers and practitioners will help to



For peer review only - http://bmjopen.bmj.com/site/about/guidelines.xhtml

explore options for scale-up of a sustainable model. Few studies have explored current early child-care practices, the need and demand for day-care in Bangladesh or other low and lower-middle-income countries. This study should provide useful insights to those in research and practice who are keen to identify effective and appropriate day-care models.

As described above, there are currently a plethora of tools to assess ECD. There have been recent attempts to synthesise ECD tools, resulting in the development of MELQO [30] This is timely given that Sustainable Development Goal (SDG) Target 4.2, which aims that, by the year 2030, 'all girls and boys have access to quality ECD , care and pre-primary education so that they are ready for primary education'[6]. Our findings will contribute to the knowledge base of the feasibility and acceptability of these tools in an urban low-income context.

A limitation of our study is that, due to the resources available, we are only able to pilot our day-care model in one community. Clearly, insights from different types of urban neighbourhoods would have been valuable in informing the scale up of the day-care model. Similarly, our questionnaire is conducted only one ward of Dhaka. While our sample size is sufficiently powered to provide estimations of demand for day-care, the external validity of the questionnaire to infer to other types of neighbourhoods within Dhaka and other cities in low-income countries is low.

The purpose of this paper is to share the study protocol for developing sustainable day-care centres for 1-4 year olds in Bangladesh. This paper should be of interest to researchers interested in methodologies for assessing procedures for setting up sustainable models of day-care for under-five in urban slums. Policymakers and practitioners may also value a sustainable day-care model characterised by a strong sense of community ownership which is challenging in a mobile population. We plan to collaborate with multiple stakeholders and produce a day-care model based



For peer review only - http://bmjopen.bmj.com/site/about/guidelines.xhtml

on principles of child development with provision of nutritious meals, hygiene and education for children aged 1-4 years.

This intervention has the potential to improve a number of child health and ECD outcomes. During the study, we will identify the most appropriate primary outcome that is important to policy makers, feasible to measure and likely to be sensitive to the effects of the intervention. The outcomes we expect are:

1) Improved ECD: including physical, socio-cognitive, language and learning development, facilitated by inputs from trained childcare workers and their advice to care-givers.

2) Reduced accidents and injuries: being in well-supervised safe day-care will reduce exposure to accidents and injuries. Risk reduction will also be part of advice given to care-givers.

3) Reduced stunting (linear growth retardation): through i) improved nutrition due to provision of healthy meals during day-care and care-giver advice and ii) reduced gastro-intestinal disease due to improved sanitation, provision of clean water and handwashing education for children in day-care.

Financial sustainability is key for longevity and scale-up. This approach will promote integration between poorer and better-off families, potentially facilitating social mobility for slum children. The intervention will draw on the WHO/UNICEF evidence-based model of Care for Child Development [1, 31] which emphasises play and communication activities.

# 5 Collaborations

This project is led by Dr Elsey (UoL) who has led a number of public health intervention development, feasibility and evaluation studies. She brings expertise in public health intervention development, cross-sectional surveys, qualitative methods and PAR. Dr Das (Research Fellow) has a research experience of working in slums in low-income settings in South-East Asia and skills in



For peer review only - http://bmjopen.bmj.com/site/about/guidelines.xhtml

qualitative research. Dr Hicks brings statistical expertise. All have experience of supporting research with partners from low income settings.

CIPRB and ARK Foundation are health research NGOs with impressive portfolios of public health research and strong links to the government and health sector in Bangladesh. Dr Mashreky (CIPRB) has extensive experience of conducting household surveys. Over the last 10 years, he has led the design, implementation and evaluation of the day-care model in rural Bangladesh. CIPRB provide a strong research environment with the infrastructure required to conduct large cross-sectional studies and carefully implement the day-care model, with PAR expertise. Dr Wallace provides additional advice on injuries. Dr Huque and ARK Foundation are long-standing partners with UoL through the DFID-funded COMDIS-HSD consortium. They bring well developed qualitative skills, and strong links with local and national government. Our collaboration includes key players in ECD: Save the Children, Aga Khan Foundation and UNICEF, all of whom attended preliminary workshops in Dhaka. Save the Children will provide in-kind advisory support on ECD and training on the IDELA tool.

# 6 Authors' contributions

HE, MS, RH and HW conceived of the study. RS, JH, JF, RH, FF, SN, HE a MS developed the quantitative and qualitative data collection tools and methods. RS, JH, MS, JF and HE developed the quantitative analysis plan. HE, FF, RH developed the ethical approval forms for the ethics committees in Bangladesh and Leeds, Uk. MD drafted the manuscript with inputs from HE. All authors contributed to refinement of the paper. All authors have read and approved the final version.

# 7 Acknowledgements

This project has been funded by the MRC Public Health Intervention Development Grant: MR/P017037/1. The funding source had no role in the design and conduct of the study; collection,



For peer review only - http://bmjopen.bmj.com/site/about/guidelines.xhtml

management, analysis and interpretation of the data; preparation, review and approval of the manuscript; and decision to submit the manuscript for publication.

# 8 Competing interests

None declared.

# 9 DATA SHARING STATEMENT

Our manuscript describes a study protocol. As such, we cannot elaborate on additional unpublished data.

# 10 FIGURE LEGENDS

Figure 1: Causal pathways perpetuating poor Early Childhood Development among disadvantaged urban 1-4 olds.

Figure 2: Theory of change: Day care in urban poor neighbourhoods.



For peer review only - http://bmjopen.bmj.com/site/about/guidelines.xhtml

# 11 References


1. UNHABITAT, *State of the world's cities 2006/7.* UN-Habitat: London, UK., 2007
2. Walker, S.P., et al., *Inequality in early childhood: risk and protective factors for early child development.* Lancet, 2011. **378**(9799): p. 1325-38.
3. Nair, M.K. and S.R. Radhakrishnan, *Early childhood development in deprived urban settlements.* Indian Pediatr, 2004. **41**(3): p. 227-37.
4. WHO, *Global report on urban health: equitable healthier cities for sustainable development*, 2016: Geneva.
5. World-Population-Review, 2018, World Popluation Review.
6. UNDESA, *World Urbanization Prospects: The 2014 Revision*, 2015, United Nations Department of Economic and Social Affairs/Population Division
7. Angeles, G., et al., *The 2005 census and mapping of slums in Bangladesh: design, select results and application.* International Journal of Health Geographics [Electronic Resource], 2009. **8**: p. 32.
8. Hamadani, J.D., et al., *Use of family care indicators and their relationship with child development in Bangladesh.* Journal of Health, Population & Nutrition, 2010. **28**(1): p. 23-33.
9. Rahman, F., et al., *Cost-Effectiveness of an Injury and Drowning Prevention Program in Bangladesh.* Pediatrics, 2012. **130**(6): p. e1621-e1628.
10. Shonkoff, J.P. and A.S. Garner, *The Lifelong Effects of Early Childhood Adversity and Toxic Stress.* Pediatrics, 2011.
11. Pathey, P., *Multiple Indicator Cluster Survey* 2009.
12. Vegas, E.S., L., *The Promise of Early Childhood Development in Latin America and the Caribbean*. 2010: The World Bank.
13. TheWorldBank., *Investing in Early Childhood Development- reveiw of the World Bank*, 2015.
14. Heckman, J.J., *The Case for Investing in Disadvantaged Young Children*. In Big Ideas for Children: Investing in Our Nation's Future, ed. First Focus. 2008a: Washington, DC: First Focus.
15. Naudeau, S.M., Sebastian; Premand, Patrick; Filmer, Deon *Cognitive Development among Young Children in Low-income countries*. No Small Matter: The Impact of Poverty, Shocks, and Human Capital Investments in Early Childhood Development,, ed. H. Alderman. 2011: World Bank.
16. Heckman, J.J.M.V., *The Productivity Argument for Investing in Young Children.* Review of Agricultural Economics, 2007. **29, number 3**: p. 446-493.
17. Moss, P., G. Dahlberg, and A.Pence. , *Getting beyond the Problem with Quality.* European Early Childhood Education Research Journal 2000. **8 (2)** p. 103-15.
18. Engle, P.L., et al., *Strategies for reducing inequalities and improving developmental outcomes for young children in low-income and middle-income countries.* Lancet, 2011. **378**(9799): p. 1339-53.
19. Mashreky, S.R., et al., *Epidemiology of childhood burn: Yield of largest community based injury survey in Bangladesh.* Burns, 2008. **34**(6): p. 856-862.




For peer review only - http://bmjopen.bmj.com/site/about/guidelines.xhtml

20. Brown, T.W., et al., *Centre-based day care for children younger than five years of age in low- and middle-income countries.* Cochrane Database of Systematic Reviews, 2014. **9**: p. CD010543.
21. Leroy, J., P,Gadsden, and M,Guijarro., *The Impact of Daycare Programs On Child Health, Nutrition And Development In Developing Countries: A Systematic Review 3iE.* The International Initiative for Impact Evaluation (3ie). 2011.
22. Pisani, L., I. Borisova, and A. J. Dowd., *International Development and Early Learning Assessment Technical Working Paper*, 2015, Save the Children Fund.
23. GoB, *Comprehensive Early Childhood Care and Development Policy*, 2013, Ministry of Women and Children Affairs (MOWCA), Government of Bangladesh.
24. *Implementation research toolkit : participants manual.* World Health Organization (WHO), 2014.
25. Jin, X., et al., *"Care for Development" Intervention in Rural China: A Prospective Follow-up Study.* Journal of Developmental & Behavioral Pediatrics, 2007. **28**(3): p. 213-218.
26. Rahman, A., et al. *Mothers' mental health and infant growth: a case-control study from Rawalpindi, Pakistan.* 2004 [cited 2018 25 April]; 1:[21-27].
27. Creswell, J.W., Klassen, A.C., Clark V.L.P., Smith K.C. , *Best Practices for Mixed Methods Research in the Health Sciences* in *The Nature and Design of Mixed Methods Research*2011 OBSSR.
28. Reason, P., Bradbury,H. , *The SAGE Handbook of Action Research Hardcover*, ed. H. Bradbury-Huang. 2015 Sage
29. McCoy DC, S.C., Bellinger DC, Muhihi A, Ashery G, Weary TE, Fawzi W, Fink G. *Development and validation of an early childhood development scale for use in low-resourced settings.* Popul Health Metr 2017 [cited 2018 4 May].
30. UNESCO- UNICEF., B.I., World Bank. *Overview MELQO - Measuring Early Learning Quality and Outcomes.* 2017.
31. Brinkman, S.A., et al., *The role of preschool quality in promoting child development: evidence from rural Indonesia.* European Early Childhood Education Research Journal, 2017. **25**(4): p. 483-505.
32. Bradbury, H. and C. Mainemelis, *Learning History and Organizational Praxis.* Journal of Management Inquiry, 2001. **10**: p. 340.
33. Ritchie J, L.J., *Qualitative research practice: a guide for social science students and researchers.* 2003, London: : Sage.
34. Ritchie J., S., L., *Qualitative data analysis for applied policy research*, in *Analyzing qualitative data.* 1994. p. 173-194.




For peer review only - http://bmjopen.bmj.com/site/about/guidelines.xhtml

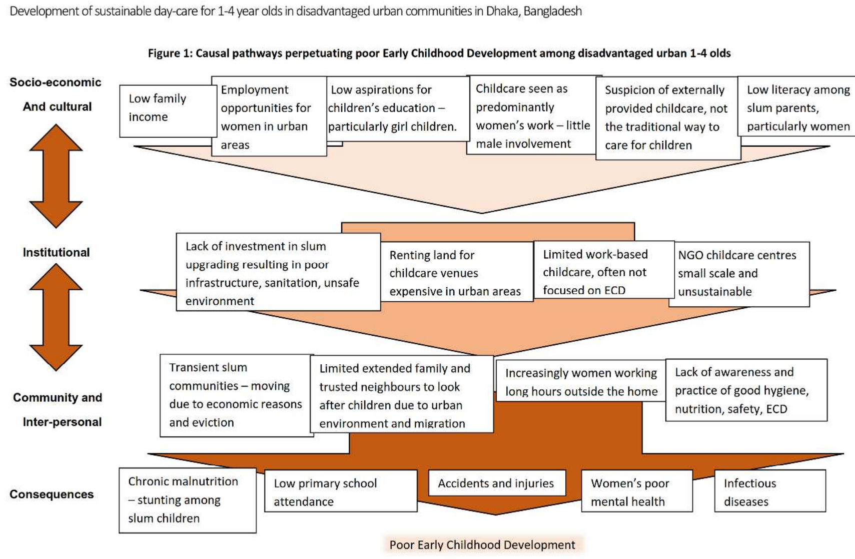


Figure 1 : causal pathways perpetuating poor early childhood development among disadvantaged urban 1-4 year olds

297x209mm (300 x 300 DPI)

ew only

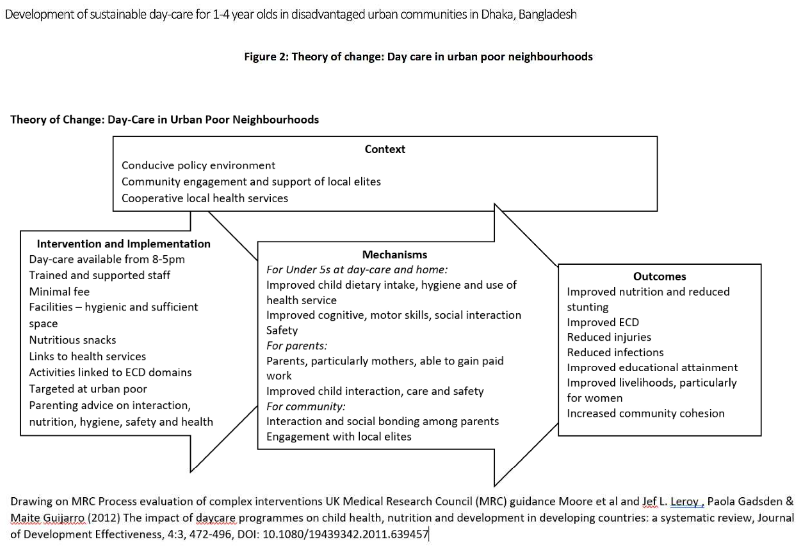


Figure 2: Theory of change: Day care in urban poor neighbourhoods.

297x209mm (300 x 300 DPI)

iew only
