# Supplementary material for: Protocol to develop sustainable day care for children aged 1–4 years in disadvantaged urban communities in Dhaka, Bangladesh
Source: BMJ Open. 2018 Aug 1;8(7):e024101. doi: 10.1136/bmjopen-2018-024101 (PMC6074616; doi:10.1136/bmjopen-2018-024101)
Supplement: Reviewer comments [file bmjopen-2018-024101.reviewer_comments.pdf]

PEER REVIEW HISTORY

BMJ Open publishes all reviews undertaken for accepted manuscripts. Reviewers are asked to complete a checklist review form (**http://bmjopen.bmj.com/site/about/resources/checklist.pdf**) and are provided with free text boxes to elaborate on their assessment. These free text comments are reproduced below.

ARTICLE DETAILS

**TITLE (PROVISIONAL)**	A protocol to develop sustainable day-care for 1-4 year olds in disadvantaged urban communities in Dhaka, Bangladesh
**AUTHORS**	Das, Mahua; Elsey, Helen; Shawon, R.A; Hicks, Joseph; Ferdoush, J; Huque, Rumana; Fieroze, Fariza; Nasreen, Shammi; Wallace, Hilary; Mashreky, Saidur

This article was not externally reviewed at BMJ Open. Protocols that have been independently assessed prior to submission to BMJ Open are usually fast-tracked to publication on the grounds that further substantial changes will not be possible. This independent assessment will usually be external, independent review for both a substantial grant award from a non-commercial or government funder and ethics approval.
